# Synopsis of the Methods of Treating Asiatic Cholera

**Published:** 1849-04

**Authors:** 


### SELECTIONS

FROM

### AMERICAN AND FOREIGN JOURNALS.

---

*Synopsis of the Methods of Treating Asiatic Cholera.* DR. GRAVES: No faith in mercury. A scruple of acetate of lead with one grain of opium, divided into twelve pills; one to be given every half hour till the discharges diminish.—*Clinical Lectures*, 2d Ed. vol. i, p. 419.

2. DR. WOOD, Philadelphia: Calomel and opium, in small, repeated doses; acetate of lead, kino; cold water to drink; external warmth; diffusible stimulants.—*Treatise on the Practice of Medicine*, vol. i.

3. DR. PARKES: First stage, bloodletting sometimes; acetate of lead, two to three grains, with a quarter grain of opium, every half hour for two or three hours; external warmth useless; large doses of calomel injurious; mustard poultices to epigastrium; cold drinks; diffusible stimuli. In *collapse*, bloodletting sometimes relieves.—No treatment to be relied upon.—*Researches on Algide Cholera.*

4. DR. MILROY: External warmth; saline emetics, as salt, one tablespoonful in a tumbler of water; turpentine stupe; salt or turpentine enemata; calomel when the vomiting has abated.—*Pamphlet on Quarantine*, 1847.

5. MR. BELL: Bloodletting, if seen in three or four hours from invasion; quinæ disulph. grs. xij; ferri sulph. grs. ix; aquæ Oiss. Dose not stated.—*Medical Gaz.* Jan. 1848.

6. Dr. Black: Small bleeding in stout subjects; calomel and croton oil repeated three times; then calomel with capsicum; enemata of warm water.—*Prov. Med. and Suug. Jour.*, Jan. 26, 1848.

7. Dr. King: Cold water *ad libitum;* large doses of calomel.

8. Dr. Turnbull: Capsicum embrocations.—*Lancet*, Jan. 29, 1848.

9. Dr. Arthur Wilson: Warm mustard emetic; venesection where possible; neutral non-aperient alkaline salts; inhalation of oxygen.—*Lancet*, Nov. 4, 1848.

10. Dr. Ayres: Two grs. of calomel and two drops of laudanum every ten minutes, as long as collapse lasts. —*Lancet*, Oct. 7, 1848.

11. Dr. Henriques: Quinine in large doses, in all stages; stimulant embroctions; injections of decoction of bark.

12. Mr. Allen: Large doses of calomel at the commencent; bleeding occasionally; mustard poultices to the spine and abdomen; enemata of hot salt and water.—*Lancet*, Oct. 21, 1848.

13. Dr. M'Cann: Mustard emetic; brandy and laudanum, and calomel and opium; stimulant embrocotions.—*Lancet*, Oct. 21.

14. Mr. Hird: Mustard emetics, followed by acetate of lead and opium; stimulating apothems.—*Lancet*, October 21.

15. Mr. Jenkins: Strychnia, gr. j; conserve of roses sufficient to form eighteen pills; one every quarter of an hour.

16. Mr. Beaman: Salt emetics; external warmth; then carbonate of soda in effervescence with lemon juice.—*Lancet*, Sept. 2.

17. Mr. Hancorn: Emetics; diffusible stimulants, as ammonia, capsicum; hyd. c. creta; tinct. ferri sesquichloridi in concentrated form after every motion; sulphuric acid embrocations; hot-air bath.—*Lancet*, Sept. 9, 1848.

18. Dr. Radcliffe Hall: Five grs. of tartar emetic in half a pint of camphor mixture; an ounce every two hours, till tolerance is effected.

19. MR. BRADY: In premonitory stage, ol. ricin. ʒiij; chloroform, ♏vj; tinct. opii, ♏xx; aquæ menthæ, ℥iss; f. haust. If reaction ensues, external warmth, sinapisms, and the following draught and pill, repeated according to circumstances:

℞. Chloroformi, ♏viij;

Sp. vin. gall., ʒiij.

Aquæ, ℥ij. f. Haust.

℞. Fellis bovini, grs. iv;

Hyd. chlor., grs. iij. f. Pil.

Chloroform embrocations to the spine.—*Medical Times*, Oct. 14.

20. DR. SHEARMAN: Bloodletting, followed by transfusion of blood; respiration of oxygen and atmospheric air; tartar emetic treatment.—*Medical Gazette*, October 14, 1848.

21. DR. PATTERSON, Rathkeale: Five grains of calomel, with thirty drops of laudanum, every four hours; then an enema, consisting of sulphate of copper, sulphate of zinc, and alum, a scruple of each, to one ounce of cold water; a wineglassful thrown up every few minutes till retained; after retention for half an hour, a large warm water injection.—*Dublin Med. Press*, Sept. 20.

22. DR. COWAN: Thinks well of bleeding in robust persons; stimulating emetics; calomel and opium; effervescing salines *ad libitum;* external warmth.—*Prov. Med. and Surg. Jour.*, Nov. 1, 1848.

23. SIR JAMES MURRAY: A wineglassful of his fluid of camphor every ten minutes, with a few drops of laudanum, inflating the lungs with electrified air; galvanic discharges through the respiratory and spinal nerves.—*Lancet*, Nov. 4, 1848.

24. MR. MARSDEN: Calomel and ginger, with powders of common salt, ʒij, carbonate of soda, ℈j, oxymuriate of potassa, grs. vij, every quarter of an hour till reaction ensues; hot salt baths; warm saline emetics.—*Lancet* Nov. 4, 1848.

25. Dr. Willemin and M. Moreau: Cannabine, the active principle of Indian hemp. The preparation a tincture of the strength of one grain to ten drops of alcohol; dose, ten to fifteen drops.—*Lancet*, November 4, 1848.

26. Dr. Hill: Place the patient in a warm bed; give internal stimulants; friction with warm flannels; external heat; chloroform inhalations repeated at intervals.—*Lancet*, Nov. 4, 1848.—*Ranking's Abstract.*

